# Diagnostic Accuracy of Electro-Chemiluminescence Immunoassay Anti-SARS-CoV-2 Serological Test

**DOI:** 10.7759/cureus.12588

**Published:** 2021-01-09

**Authors:** Nayab Afzal, Naila Tariq, Saba Raza, Danish Shakeel

**Affiliations:** 1 Clinical Chemistry, National Medical Center, Karachi, PAK; 2 Chemical Pathology, National Medical Center, Karachi, PAK; 3 Clinical Chemistry, Ziauddin University, Karachi, PAK; 4 Hematology, National Medical Center, Karachi, PAK

**Keywords:** covid 19, anti-sars-cov-2 antibodies, diagnostic accuracy, rt-pcr

## Abstract

Objective: To determine the diagnostic accuracy of fully automated electro-chemiluminescence immunoassay (ECLIA) anti-SARS-CoV-2 serological test for detection of past SARS-CoV-2 infection and to be used in seroprevalence surveys.

Method: A total of 426 patients who had tested for anti-SARS-CoV-2 from August 1 to 31, 2020 were selected for the study. Informed consent was obtained and a questionnaire including the patient’s age, gender, symptoms, and past polymerase chain reaction (PCR) status was filled by the patient. Samples were analyzed for anti-SARS-CoV-2 antibodies on Roche Cobas e601.

Results: The mean age of the patients was 42.43±16.67 years. One hundred and five (24.6%) were PCR positive, while 321 (75.4%) were PCR negative. Most patients were males 241 (56.6%) while 185(43.3%) were females. Over 185(43.3%) patients presented with symptoms, and the rest of the patients 241 (56.6%) were asymptomatic. Anti-SARS-CoV-2 had sensitivity 89.5%, specificity 99.06%, positive predictive value (PPV) 96.90%, negative predictive value (NPV) 96.6%, and positive likelihood ratio 4.26, while negative likelihood ratio 0.1. Diagnostic accuracy of anti-SARS-CoV-2 was 96.7% based on receiver-operating characteristic (ROC) curve analysis.

Conclusion: Anti-SARS-CoV-2 is very useful for the detection of past COVID-19 infection; it can be proved helpful in the identification of post-COVID complications and actual disease burden in a population.

## Introduction

Towards the end of 2019, the Chinese city of Wuhan reported a cluster of pneumonia patients [1]. Upon thorough laboratory analysis of bronchoalveolar lavage fluid of these patients, a novel Coronavirus was identified. This new organism was named severe acute respiratory syndrome Coronavirus 2 (SARS-CoV-2) and the disease was called Coronavirus disease 2019 (COVID-19) [2,3].^ ^Soon the world was grappling with a pandemic of massive proportions. Until now this disease has more than 27 million confirmed cases and about 890 thousand people have lost their lives in this pandemic [4].

Whenever the scientific community has come face to face with a new infection knowledge regarding the source of the pathogen, mode of transmission, life cycle, pathogenicity, diagnostic tools, and treatment protocols are gradually perfected over a period of years. Because of the unprecedented rapid spread of COVID-19, the healthcare systems around the world were presented with many clinical and diagnostic challenges. Several newly developed screening and diagnostic test techniques started to fulfill the insatiable demand for rapid diagnosis across the globe. Real‐time reverse‐transcription polymerase chain reaction (RT‐PCR) which detects SARS‐CoV‐2 in the throat and nasal swabs became the golden standard for diagnosis of COVID-19 [5]. The performance of RT-PCR was influenced by several factors like sample collection skill, the interval between sample collection and appearance of symptoms, viral load, faulty sample processing, and quality of RT-PCR assay being used. Additionally, only symptomatic patients were advised RT-PCR, missing out on the asymptomatic population. As more and more studies came out regarding asymptomatic cases and patients presenting first time with complications of COVID-19 like myocardial infarction, stroke, acute kidney injury, hepatocellular injury, and thrombotic complications also started to be documented [6-9]. This further intensified the need for a rapid, easy, sensitive, and specific investigation for the identification of such individuals adding a layer of complexity to an already perplexing situation.

Rapid serological test kits identifying different antibodies (IgM, IgG) generated a lot of interest because of their faster turn-around time, simplicity, and cost. However, the clinical worth of these tests depends mainly upon the performance characteristics like sensitivity, specificity, positive predictive value, and negative predictive value. The lack of adequate vigilance of serologic tests is concerning given that the commercially available serologic assays are highly variable, having different principles, detect variable antibody class, and have inconsistent sensitivities. Many rapid testing kits were based on lateral-flow immunoassay (LFIA) or enzyme-linked immunosorbent assay (ELISA) technique which have questionable results and have been called back from many centers [10]. An added problem is the lack of a deep understanding of how these assays work and more importantly what exactly they indicate.

Recently, fully automated electro-chemiluminescence immunoassay (ECLIA) has been introduced claiming to be highly sensitive and specific. The aim of our study is to determine the diagnostic accuracy of anti-SARS-CoV-2 (ECLIA technique) for diagnosis of past infection with COVID-19 using RT-PCR as the golden standard.

## Materials and methods

This cross-sectional study was conducted from August 1 to 31, 2020 at the Clinical Chemistry section of National Medical Center Laboratory Karachi. A total of 426 consenting patients who visited the outpatient or emergency department of the hospital were included in this study. The study purpose was thoroughly explained to the patients. For all the enrolled patients, a questionnaire regarding age, gender, symptoms (dry cough, fever, shortness of breath, sore throat, loss of taste/smell, headaches, body aches, diarrhea, and vomiting), and RT-PCR result for COVID-19 (report presented by the patient) in past 15-21 days were filled by the patient.

Venous blood samples were collected using a serum separator tube (SST). Tubes were centrifuged at 4000*g* for 15 minutes to separate the serum. All antibody tests were performed using a fully automated Roche Cobas e601 immunoassay analyzer based on the ECLIA technique. This anti‑SARS‑CoV-2 assay uses a recombinant protein representing the nucleocapsid (N) antigen for the determination of antibodies (total, both IgM and IgG) against SARS‑CoV‑2. The calibration and internal quality control were done according to the manufacturer’s recommendations.

Patient samples were labeled as reactive (cut off >1.000) and non-reactive (cut off <1.000) as recommended by the manufacturer. Data were organized and entered on SPSS version 22 (IBM Corp., Armonk, New York). Age was presented as mean ± standard deviation. The frequency of symptoms, gender, and history of positive or negative RT-PCR was calculated with Pearson's co-efficient p-values <0.05 taken as statistically significant.

Diagnostic accuracy was presented in terms of sensitivity, specificity, positive and negative predictive values, positive and negative likelihood ratios. ROC curve analysis was done to validate the performance of the anti-SARS-CoV-2 test.

## Results

The average age of the patients was 42.43±16.67 years. The largest proportion of patients belonged to the 21-40 years age group 42.7% (n=182). Males constituted 56.6% (n=241) of the study group. The majority of patients were asymptomatic and tested negative for COVID-19 RT-PCR.

Fever was noted to be the commonest symptom followed by body aches and sore throat as shown in Table 1.

**Table 1:** Frequency of different symptoms (n=185)

Symptoms	No. of cases	Percentage (%)
Fever	91	21.4
Body aches	50	11.7
Sore throat	39	9.2
Cough	31	7.3
Shortness of breath	30	7.0
Loss of smell	27	6.3
Vomiting	16	3.8
Loss of taste	13	3.1
Headache	13	3.1
Diarrhea	10	2.3

Stratification analysis was performed and it was observed that the frequency of anti-SARS-CoV-2 reactivity was significantly high in people who were symptomatic or had COVID-19 RT-PCR positive in the past 14-21 days (p-value <0.05). However, no statistically significant difference was found in regard with age groups or gender (p-value >0.05), as represented in Table 2.

**Table 2:** Frequency of age groups, gender, symptoms, and PCR results (n=426)

Variable	Options	No. of cases	Percentage (%)	p-Value
Age (years)	0-20	34	8	0.989
	21-40	182	42.7	
	41-60	144	33.8	
	>60	66	15.5	
Gender	Males	241	56.6	0.686
	Females	185	43.3	
Symptoms	Symptomatic	185	43.4	<0.001
	Asymptomatic	241	56.6	
PCR results	Positive	105	25.4	<0.001
	Negative	321	74.6	
Symptoms in PCR positive cases	Yes	97	92.4	<0.001
	No	07	7.6	

Anti-SARS-Cov-2 was found reactive in 22.8% (n=97) patients while 72.2% (n=329) were found non-reactive. Sensitivity, specificity, PPV, NPV, positive likelihood ratio, negative likelihood ratio, and diagnostic accuracy are excellent as shown in Table 3.

**Table 3:** Diagnostic accuracy of anti-SARS-CoV-2 in the detection of past COVID-19 infection taking RT-PCR as the golden standard Sensitivity = TP/(TP+FN); Specificity = TN/(TN+FP); PPV=TP/(TP+FP); NPV  = TN/(TN+FN); Likelihood ratio positive = sensitivity/(100 − specificity); Likelihood ratio negative = (100 − sensitivity)/specificity; Diagnostic accuracy = (TP+TN)/(TP+TN+FP+FN); PPV: positive predictive value; NPV: negative predictive value; POS-LR: positive likelihood ratio; NEG-LR: negative likelihood ratio; Diag accuracy: diagnostic accuracy.

Anti-SARS CoV-2 antibody	RT-PCR for COVID-19		Total
	Positive	Negative	
Reactive	94 (TP)	3 (FP)	97 (22.8%)
Non-reactive	11 (FN)	318 (TN)	329 (72.2%)
Total	105	321	426
Sensitivity	=94/105	=89.5%	
Specificity	=318/321	=99.06%	
PPV	=94/97	=96.90%	
NPV	=318/329	=96.6%	
POS-LR	=89.5/100-99.06	=95.21	
NEG-LR	=100-89.5/99.06	=0.1	
Diag accuracy	=94+318/426	=96.7%	

**Figure 1:**
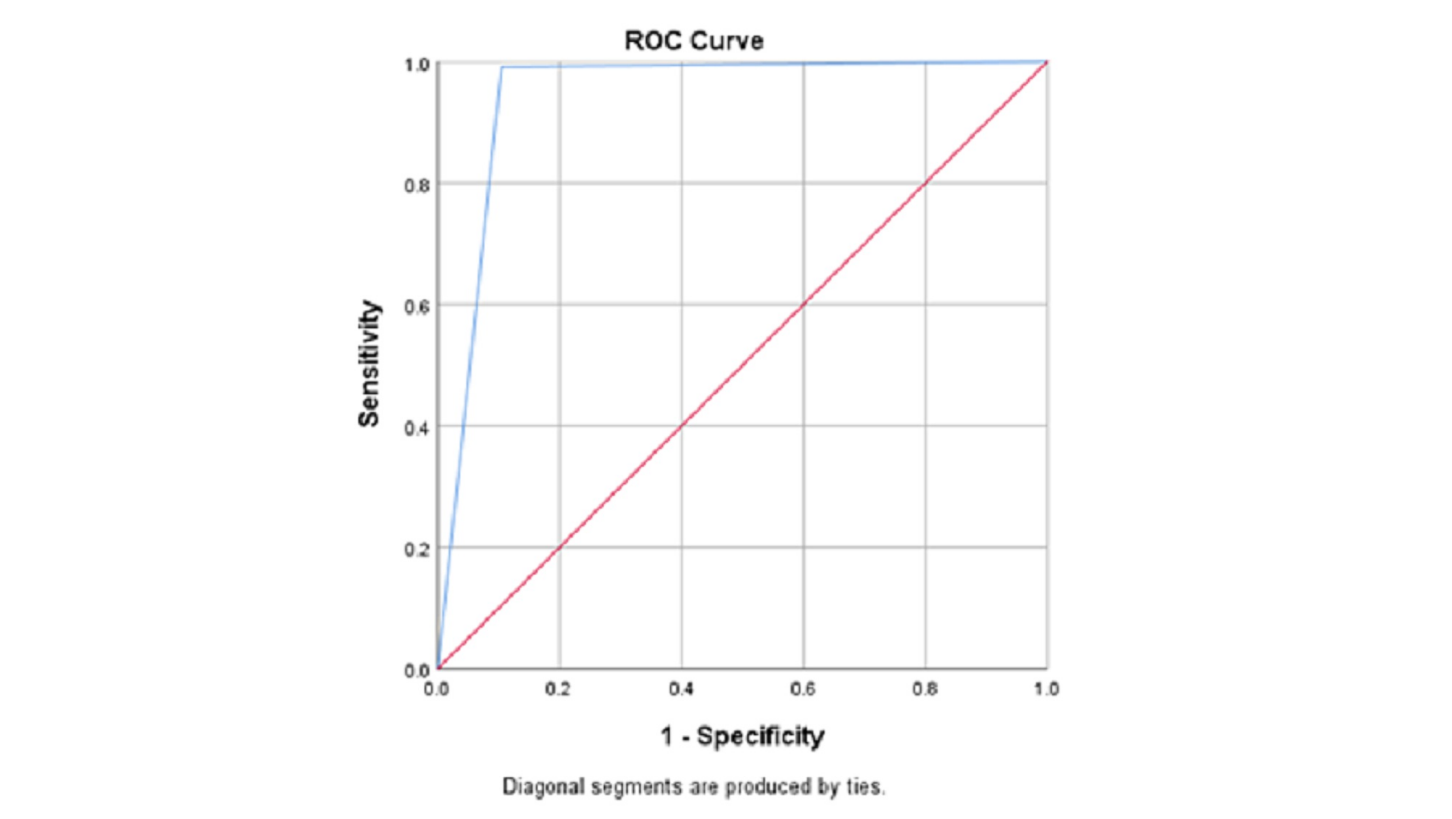
ROC-AUC analysis of anti-SARS-COV-2 serological test

The area under the ROC curve (AUC) for anti-SARS-Cov-2 was 0.943 which is considered an excellent value, as depicted in Figure 1.

## Discussion

Serum antibody tests are not for the detection of the presence of virus instead they tell us about the immunoglobulins (Ig) formed by B lymphocytes against the virus in the human body as a protective mechanism [11]. With the fast spread of COVID-19, various tests were hurriedly marketed for detection of the antibodies produced in response to this virus. These tests can be broadly classified into manual and automated methods. Manual testing kits (cassettes and cartridges) made use of LFIA or lateral-flow immunochromatography technique that relies on color change visible to the naked eye after the antigen-antibody reaction. These tests are quick and easy to perform but the results can be highly variable [12]. However, the high sensitivity and specificity of serological tests are of utmost importance for appropriate patient evaluation and management. The rapid influx of multiple testing techniques and platforms has resulted in a lot of confusion among treating physicians. Assays based on the ECLIA technique claim to be highly sensitive and specific leading to better diagnostic accuracy [13].

The total number of patients with positive PCR results was 105, out of which 97 (92.4%) patients presented with symptoms, the rest of the patients 07 (7.6%) were asymptomatic. These results are similar to another study from Karachi which showed only 9.0% were asymptomatic [14]. A significant association was found between anti-SARS-CoV-2 reactivity appearance of symptoms and RT-PCR positivity; however, no association could be established with age or gender. Similar findings were reported by Anand et al. [15]. In the current study, fever (21.4%) was found to be the commonest symptom followed by body aches (11.7%), on the other hand, fever was present in 83.3% of patients and 38% of people complained of body aches in a study carried out in China [16]. These differences can be due to variation in the humoral response and immunity status of different populations making the symptoms variable.

In this study, anti-SARS-CoV-2 misidentified only 0.028% as false positives, while 99.06% were correctly classified as true negative, i.e., these patients also had negative PCR results, making this test highly specific. It is important to have very high specificity in order to minimize the risk of cross-reactivity with other viruses that cause common cold or influenza. The sensitivity of this test was found to be 89.5%. This slight low sensitivity may be attributed to variation in immune response and timings of sample collection. These observations differ from a study conducted by Muench et al. which showed better sensitivity and specificity of 99.5% and 99.08%, respectively. The stark gap in the noted sensitivity of this test calls for further validations [17].

The high negative predictive value in this study provides a strong reassurance that a person has not suffered from COVID-19 in the near past. The sensitivity, specificity, PPV, NPV values, and accuracy of anti-SARS-CoV-2 were consistent with a study done by Lau et al. [18]. AUC is an efficient and effective way to identify the diagnostic accuracy of the test in question. AUC value in our study came out to be 0.943, which suggests a 94.3% chance that the anti-SARS-CoV-2 test will correctly distinguish a person with past COVID-19 infection from a person who did not get infected with COVID-19.

Given the excellent performance characteristics of anti-SARS-CoV-2, it can be confidently used as a tool to assess community exposure and can help in making future health policies.

However, the results of the current study can be improved upon by multicentre data collection and by longer duration for studying the performance of this test. In addition to this, the anti-SARS-CoV-2 test used in this study is approved only for qualitative analysis of antibodies which is superseded by quantitative assays helping in improved reporting in COVID 19 patients.

## Conclusions

Our study points towards high sensitivity, specificity, PPV, NPV, and excellent diagnostic accuracy of fully automated ECLIA anti-SARS-CoV-2 method, making it an ideal choice for identification of past COVID 19 infections and can be used as an efficient tool for mass screening and seroprevalence studies been done for detection of actual COVID-19 burden in a population.

In conclusion, we can say that such a serological assay to detect antibodies against SARS-CoV2 can be used as an effective tool in our current response to the COVID-19 pandemic. As time progresses, we will learn more about our immune response to this virus, duration, and level of protective immunity provided by the development of antibodies against SARS-CoV-2. With existing knowledge about the advantages and limitations of this serological test, it can be safely recommended for use in public health studies and academic proposes, but additional data are required to build up on this foundation.
